# Impact of persistent D-dimer elevation following recovery from COVID-19

**DOI:** 10.1371/journal.pone.0258351

**Published:** 2021-10-28

**Authors:** Antje Lehmann, Helmut Prosch, Sonja Zehetmayer, Maximilian Robert Gysan, Dominik Bernitzky, Karin Vonbank, Marco Idzko, Daniela Gompelmann

**Affiliations:** 1 Department of Medicine II, Division of Pulmonology, Medical University of Vienna, Vienna, Austria; 2 Department of Biomedical Imaging and Image-guided Therapy, Medical University of Vienna, Vienna, Austria; 3 Center for Medical Statistics, Informatics, and Intelligent Systems, Medical University of Vienna, Vienna, Austria; Al-Azhar University, EGYPT

## Abstract

**Background:**

Elevated D-dimer is known as predictor for severity of SARS-CoV2-infection. Increased D-dimer is associated with thromboembolic complications, but it is also a direct consequence of the acute lung injury seen in COVID-19 pneumonia.

**Objectives:**

To evaluate the rate of persistent elevated D-dimer and its association with thromboembolic complications and persistent ground glass opacities (GGO) after recovery from COVID-19.

**Methods:**

In this post hoc analysis of a prospective multicenter trial, patients underwent blood sampling, measurement of diffusion capacity, blood gas analysis, and multidetector computed tomography (MDCT) scan following COVID-19. In case of increased D-dimer (>0,5 μg/ml), an additional contrast medium-enhanced CT was performed in absence of contraindications. Results were compared between patients with persistent D-dimer elevation and patients with normal D-dimer level.

**Results:**

129 patients (median age 48.8 years; range 19–91 years) underwent D-Dimer assessment after a median (IQR) of 94 days (64–130) following COVID-19. D-dimer elevation was found in 15% (19/129) and was significantly more common in patients who had experienced a severe SARS-CoV2 infection that had required hospitalisation compared to patients with mild disease (p = 0.049). Contrast-medium CT (n = 15) revealed an acute pulmonary embolism in one patient and CTEPH in another patient. A significant lower mean pO2 (p = 0.015) and AaDO2 (p = 0.043) were observed in patients with persistent D-Dimer elevation, but the rate of GGO were similar in both patient groups (p = 0.33).

**Conclusion:**

In 15% of the patients recovered from COVID-19, persistent D-dimer elevation was observed after a median of 3 months following COVID-19. These patients had experienced a more severe COVID and still presented more frequently a lower mean pO2 and AaDO2.

## Introduction

Coronavirus disease (COVID-19) caused by the novel betacoronavirus SARS-CoV-2 (severe acute respiratory syndrome coronavirus 2) was first diagnosed in Wuhan in December 2019. The prompt outbreak and quick spread led to a worldwide COVID-19 pandemic. The majority of patients with SARS-CoV2 infection present mild to moderate symptoms including fever, headache, loss of smell and taste, cough and dyspnoea. However, the mortality among the minority of people with severe COVID-19 is high [1]. This fulminant disease include pneumonia, respiratory failure requiring intubation and ventilation, multi-organ failure and death.

D-Dimer is known as important predictor for severity and mortality of COVID-19 [2]. Elevated D-Dimer is most likely due to the acute lung injury itself or due to the increased rate of thromboembolic complications observed in patients with COVID-19.

The acute lung injury observed in COVID-19 is characterized by bilateral ground-glass opacities, crazy paving pattern and consolidations in peripheral distribution in computed tomography (CT) scans. Thereby, the extent of CT findings that seem to peak in week 2 to 3 during SARS-CoV2 infection may correlate with disease severity [3, 4]. This parenchymal involvement seems to be reflected by the D-Dimer level. Patients in whom CT scan shows consolidations and ground-glass opacities have higher D-Dimer levels compared to those without abnormalities in the CT scan [5].

Besides, the acute lung injury, SARS CoV-2 infections are associated with an increased rate of thromboembolic events resulting from an imbalance between procoagulant factors and natural coagulation inhibitors, fibrinolysis shutdown, endothelial injury and inflammatory processes [6]. Pulmonary embolism can be revealed in 20–30% of the patients with an acute COVID-19 [7, 8]. Mouhat and colleagues revealed that a D-dimer level more than 2590 ng/ml could predict the risk of pulmonary embolism, whereas Ventura and colleagues even found a higher D-dimer level (> 2903 ng/ml) to be suspicious for thromboembolic complications in patients with COVID-19 [7, 9].

It is known that 62% of patients still present CT scan abnormalities one month following COVID-19 and that 39% of patients suffer from “long COVID” with persistent symptoms 4 weeks after infection [10, 11]. However, there are only limited data about the impact of D-Dimer levels in the Follow-up of patients recovered from COVID-19. This analysis evaluated the impact of D-Dimer in patients who recovered from COVID-19 in the long-term follow-up.

## Material and methods

This post-hoc analysis of a prospective trial evaluated the impact of D-dimer level in patients who recovered from COVID-19 within the last 6 months. This study was performed, in accordance with the provisions of the Declaration of Helsinki, at the Medical University of Vienna, Hietzing Hospital Vienna and Otto-Wagner Hospital, Vienna. The protocol of this trial was approved by the ethics committee of the University of Vienna (1551/2020) and all patients gave written informed consent.

### Subject enrolment and assessment of clinical data

Patients who suffered from COVID-19 confirmed by a positive polymerase chain reaction (PCR) and recovered from infection within the last 6 months were eligible for this post-hoc analysis. Main exclusion criterion was a pre-existing concomitant lung disease such as chronic obstructive pulmonary disease, asthma or interstitial lung disease. At timepoint of study enrolment all patients underwent once a laboratory test for D-dimer level, fibrinogen, C-reactive protein (CRP), measurement of diffusion capacity (DLCO SB and DLCO/VA) and blood gas analysis. Moreover, multidetector computed tomography scan (MDCT) was taken to assess ground-glass opacities, consolidations or crazy-paving pattern that present the key features of COVID-19 pneumonia. In case of elevated D-dimer level, patients underwent contrast-medium enhanced CT scan to evaluate pulmonary embolism in absence of contraindications. The CT pulmonary angiography scans were performed on a Siemens Somatom Drive (Siemens Healthineers, Germany) scanner with a standard protocol with 50 ml contrast agent (Iomeron 400mg/ml, Bracco, Italy) followed by 40 ml saline during mild inspiratory breath hold. Images were reconstructed in coronal and axial plane with 3, and respectively 1 mm slice thickness in soft-tissue and lung window. Maximum intensity projections (MIPs, Br54 kernel) with 12 mm (3 mm increment) in soft-tissue window in axial, as well as coronal planes, for an improved detection of pulmonary thrombi, were generated.

### Statistical analysis

The sample size of this exploratory study can not be fixed in advance. We assume a sample size of 50–500 patients with the proven diagnosis of an allotted COVID-19 infection.

For data description and group comparisons absolute and % frequencies and Chi-square tests are given for categorical data, mean ± standard deviation and t-test for continuous data. Due to skewed distributions for pCO2, Fibrinogen and CRP median ± interquartile range and Mann-Whitney U-test were calculated (no test was calculated for Fibrinogen due to the small number of observations in D-Dimer>0.5 group). The significance level was set to 0.05. Due to the exploratory character of the study, no adjustment for multiplicity was performed and p-values are interpreted exploratory.

## Results

### Subject characteristics

**Fig 1:**
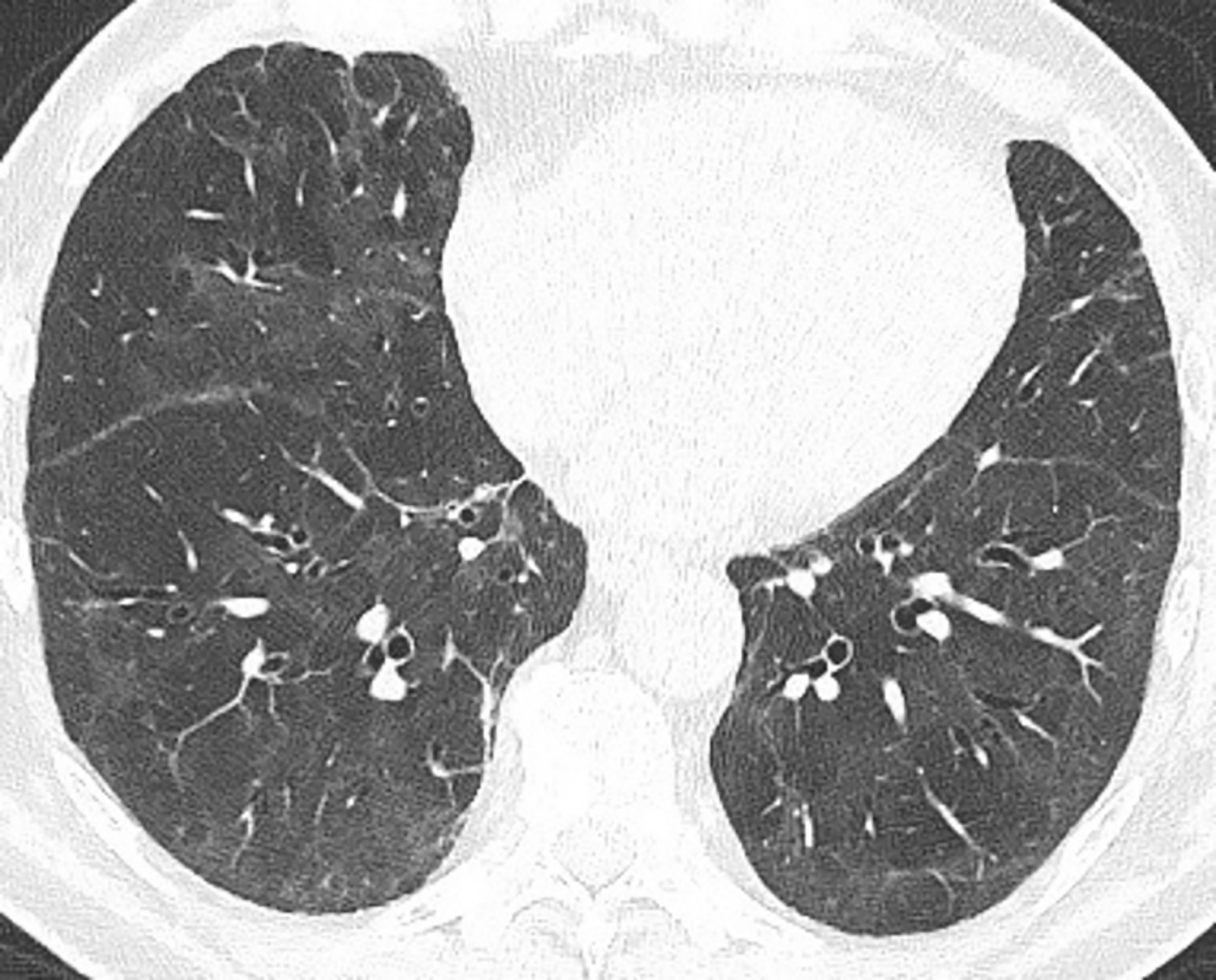
Computed tomography scan 3 months following SARS-CoV2 infection. Bilateral ground-glass opacities and mild peripheral reticular pattern.

In this analysis, 129 subjects (male 48.8%, mean age 48.9 ± 15.7 years, range 19–91 years) were enrolled after a median (IQR) of 94 days (64–130) following COVID-19 in this prospective trial. In the case history, 34.1% (44/129) and 20.9% (27/129) of the subjects reported about dyspnoea or thoracic pain respectively at the time point of SARS-CoV2 infection and 26.4% (34/129) have been hospitalized due to COVID-19. At study enrolment, mean DLCO SB was 82.6% ± 16.7% (range 35.3 to 120.5%) and mean DLCO/VA was 93.3% ± 13.7% (range 50.4 to 131.3%). Blood gas analysis revealed a mean pO2 of 89.1 ± 9.2 mmHg and a mean pCO2 of 41.1 ± 24.9 mmHg. In MDCT, ground glass opacities and consolidations were still found in 34.9% (45/129) of the cases (Fig 1). Subject characteristics are presented in Table 1. For details see S1 Dataset.

**Table 1:** Subject characteristics at study enrolment.

	Mean (median)	SD (*IQR)	min	max
**DLCO SB (mmol/min/kPa)**	7.94	2.32	2.3	14.8
**DLCO SB (%)**	82.6	16.7	35.3	120.5
**DLCO/VA (mmol/min/kPa/L)**	1.45	0.25	0.7	2.2
**DLCO/VA (%)**	93.3	13.7	50.4	131.3
**AaDO2 (mmHg)**	13.9	8.5	0.2	37.5
**pO2 (mmHg)**	89.1	9.2	66.5	109
pCO2 (mmHg)*	39.1	5.2	30	65.6
**D-dimer (μg/ml)**	0.61	2.39	0.26	27
Fibrinogen*	293	90	201	594
**IL-6 (pg/ml)**	3.31	3.3	1.53	17.7
CRP (mg/dl)*	0.12	0.2	0.3	1.3

### D-dimer elevation

At study enrolment, laboratory test that was taken after a median (IQR) of 94 days (64–130) following COVID-19 diagnosis revealed a mean D-Dimer level of 0.61 μg/ml ± 2.39 μg/ml (range 0.26 to 27 μg/ml). An elevated D-dimer level (>0,5 μg/l) was found in 14.7% (19/129). Out of these 19 subjects, 15 patients underwent contrast-medium enhanced CT scan that confirmed pulmonary embolism in 1 patient and CTEPH (chronic thromboembolic pulmonary hypertension) in another patient. CTPEH was confirmed by ventilation-perfusion-scan and right heart catheterization (mPAP 23 mmHg). In 13 patients, no thromboembolic complication was found. No contrast medium-enhanced CT was performed in 4 patients due to renal failure, already existing anticoagulation or patient´s disapproval.

### Comparison of patients according to their D-dimer level

D-dimer elevation was significantly more common in patients who had experienced a severe SARS-CoV2 infection that had required hospitalisation compared to patients with mild disease (47 vs. 23%, p = 0.049). A significant lower mean pO2 (p = 0.015) and increased alveolar-to-arterial oxygen gradient (AaDO2) (p = 0.043) were observed in patients with persistent D-Dimer elevation. DLCO SB (%) <80% was found in 71% (12/17) of patients with persistent D-Dimer level and in 64% (69/108) of patients with normal D-Dimer level. The mean DLCO SB tended to be worse in patients with elevated D-Dimer, but without statistical significance. Inflammatory markers, e.g. CRP, were higher in patients with elevated D-Dimer levels. The rate of GGO were similar in both patient groups (47% vs. 33%; p = 0.33). Table 2 presents the comparison between the patient group with elevated and normal D-Dimer level.

**Table 2:** Comparison of patients with elevated and normal D-Dimer levels following COVID-19.

	D-Dimer >0,5 μ/mL		D-Dimer≤ 0,5 μg/mL		p-value
	n = 19		n = 110		
	n		n		
**Male (%)**	19	42% (8/19)	110	50% (55/110)	0.7
**Hospitalisation in the past due to COVID 19**	19	47.4% (9/19)	110	22.7% (25/110)	0.049
**DLCO SB (%)**	17	74.7 ± 17.6	108	83.9 ± 16.3	0.057
**DLCO/VA (%)**	17	88.0 ± 16.4	108	94.1 ± 13.1	0.16
**AaDO2 (mmHg)**	18	19.5 ± 10.1	107	12.9 ± 7.9	0.015
**pO2 (mmHg)**	19	83.9 ± 12.0	107	90.0 ± 8.3	0.043
**pCO2 (mmHg)** *	19	39.1 ± 6	107	39.1± 4.7	0.42
**Fibrinogen** *	3	409 ± 285.5	46	31 ± 80.8	-
**IL-6 (pg/ml)**	3	3.8 ± 0.8	46	3.2 ± 3.5	-
**CRP (mg/dl)** *	18	0.21±0.8	109	0.11±0.13	0.007
**Ground glass opacities/consolidations in CT**	19	47.4% (9/19)	110	32.7% (36/110)	0.33

For continuous variables mean ± standard deviation are given.

*Due to skewed distributions, median ± interquartile range and Mann-Whitney U-test were calculated (no test was calculated for Fibrinogen and IL-6 due to the small number of observations in D-Dimer>0.5 group).

## Discussion

D-dimer elevation is often observed in patients with acute COVID-19 due acute lung injury itself or due thromboembolic complications that occur frequently in COVID-19. Regular screening and monitoring of D-dimer reflects disease severity and guides anticoagulation therapy.

So far, there are only limited data about the impact of prolonged D-dimer elevation in patients who recovered from an acute COVID-19 infection. One trial assessed the D-dimer level in patients at a median of 80.5 days after initial diagnosis [12]. Increased D-dimer levels were found in 25.3% of the patients, particularly in patients who had required hospital admission. Contrast-medium enhanced CT did not reveal any thromboembolic complications. However, in that study, only 21% of the patients with increased D-dimer level underwent CT pulmonary angiogram. The authors reported that however 2 patients in the elevated D-dimer group experienced subsequently vascular complications. In our analysis, the rate of increased D-dimer level with 15% was lower, but also more frequently observed in patients who had been hospitalized due to COVID-19. In our patient cohort, CT scan that was performed in 79% (15/19) of all patients with increased D-dimer confirmed thromboembolic event in 2 patients. Thus, thrombotic complications were found in 13% (2/15) of patients with prolonged elevated D-dimer level. Thus, this analysis confirms that D-Dimer level may be an important predictor for thromboembolic events in the long-term follow-up and suggest that CT pulmonary angiogram still remains an important step in the diagnostic algorithm in patients with elevated D-dimer in convalescent COVID-19, particularly in those who are still symptomatic. Earlier studies have already reported the importance of the D-Dimer as a predictor for thromboembolic events not only in patients with infectious diseases but also for example for recurrent thrombosis after withdrawal of anticoagulation therapy [13, 14].

During an acute COVID-19 pneumonia, it was shown that parenchymal involvement assessed by CT scan correlated with the D-dimer level [5]. In our analysis patients with persistent increased D-Dimer level after recovering from COVID-19 tended to present more frequently parenchymal ground-glass opacities and consolidation in the CT scan compared to patients with normal D-dimer level but the difference was not statistical significant.

Blood gas analysis demonstrated a lower mean pO2 and higher AaDO2 in patients with prolonged D-dimer elevation and thus may reflect a persistent ventilation/perfusion (V/Q) mismatch and shunting. In patients with acute COVID-19, V/Q mismatch is a key factor in the pathophysiology of hypoxemia and an increased AaDO2 is a predictor of intensive care unit admission [15, 16]. Local interstitial edema, loss of surfactant and alveolar collapse, but also the hypercoagulable state seen in COVID-19 and the development of microthrombi may explain the V/Q mismatch.

So far, the optimal thromboprophylaxis strategy in the COVID-19 patient population is uncertain and various documents have published different anticoagulation dosing strategies [17]. In studies, the risk stratification for thromboembolic complications that guides anticoagulation therapy during hospitalisation and post-discharge includes the D-Dimer measurement, as D-Dimer seems to be a predictor for thrombotic events. In our analysis, elevated D-Dimer lever was found in 19 patients. Four out of these 19 patients already received an anticoagulation treatment for atrial fibrillation. In the 2 patients in whom pulmonary embolism was detected, an anticoagulation treatment was started. However, the 13 patients with elevated D-Dimer 3 months post COVID-19 but without the evidence of thromboembolic complication did not undergo an extended anticoagulation therapy due to lack of data.

One limitation in our study is the limited size of our patient cohort. Nevertheless, the findings of this analysis evaluate the impact of D-dimer level in COVID-19 convalescence. Prolonged D-dimer elevation may assume a persistent ventilation/perfusion mismatch due to macro- and/or microthrombi and persistent inflammatory lung injury. Another limitation of this analysis is the missing D-Dimer values prior to COVID-19. Thus, elevated D-Dimer level can not only be due to COVID-19 but also due to underlying comorbidities. It is known that e.g. plasma d-dimer levels are above the normal range in patients with malignant tumors. Also in our study cohort, three patients suffered from malignant diseases of whom two presented an elevated D-Dimer level. One patient suffered from renal cell carcinoma and neurendocrin tumor, another patient from non-hodgin lymphoma. A third patient without D-Dimer elevation had a squamous cell carcinoma of the oral cavity. Therefore it is of great importance to consider underlying comorbidities as reason for elevated D-Dimer level.

This analysis confirms that D-Dimer level may be an important predictor for thromboembolic events in COVID-19 patients. In earlier studies the importance of the D-Dimer as a predictor for thromboembolic events was found not only in COVID-19 patients but also for, e.g. for recurrent thrombosis after withdrawal of anticoagulation therapy [16, 17].

Summarizing, D-dimer assessment in the long-term course of COVID-19 may have impact with respect to diagnostic and therapeutic approach of patients who recovered from COVID-19. Blood gas analysis and CT pulmonary angiogram may reveal thromboembolic complications or prolonged inflammatory processes in patients with increased D-dimer level during convalescence from COVID-19.

## Supporting information

S1 Dataset (XLSX)

## References

[1] Zhou F, Yu T, Du R, Fan G, Liu Y, Liu Z, et al. Clinical course and risk factors for mortality of adult inpatients with COVID-19 in Wuhan, China: a retrospective cohort study. Lancet. 2020;395(10229):1054–1062. doi: 10.1016/S0140-6736(20)30566-3 32171076 PMC7270627

[2] Shah S, Shah K, Patel SB, Patel FS, Osman M, Velagapudi P, et al. Elevated D-Dimer Levels Are Associated With Increased Risk of Mortality in Coronavirus Disease 2019: A Systematic Review and Meta-Analysis. Cardiol Rev. 2020 Nov/Dec;28(6):295–302. doi: 10.1097/CRD.0000000000000330 33017364 PMC7437424

[3] Zhao W, Zhong Z, Xie X, Yu Q, Liu J. Relation Between Chest CT Findings and Clinical Conditions of Coronavirus Disease (COVID-19) Pneumonia: A Multicenter Study. AJR Am J Roentgenol. 2020 May;214(5):1072–1077. doi: 10.2214/AJR.20.22976 32125873

[4] Liu H, Luo S, Zhang Y, Jiang Y, Jiang Y, Wang Y, et al. Chest CT Features of 182 Patients with Mild Coronavirus Disease 2019 (COVID-19) Pneumonia: A Longitudinal, Retrospective and Descriptive Study. Infect Dis Ther. 2020 Dec;9(4):1029–1041. doi: 10.1007/s40121-020-00352-z 33067768 PMC7566584

[5] Zhu J, Chen C, Shi R, Li B. Correlations of CT scan with high-sensitivity C-reactive protein and D-dimer in patients with coronavirus disease 2019. Pak J Med Sci. 2020 Sep-Oct;36(6):1397–1401. doi: 10.12669/pjms.36.6.2961 32968416 PMC7501002

[6] Voicu S, Delrue M, Chousterman BG, Stépanian A, Bonnin P, Malissin I, et al. Imbalance between procoagulant factors and natural coagulation inhibitors contributes to hypercoagulability in the critically ill COVID-19 patient: clinical implications. Eur Rev Med Pharmacol Sci. 2020 Sep;24(17):9161–9168. doi: 10.26355/eurrev_202009_22866 32965009

[7] Mouhat B, Besutti M, Bouiller K, Grillet F, Monnin C, Ecarnot F, et al. Elevated D-dimers and lack of anticoagulation predict PE in severe COVID-19 patients. Eur Respir J. 2020 Sep 9:2001811. doi: 10.1183/13993003.01811-2020 32907890 PMC7487272

[8] Klok FA, Kruip MJHA, van der Meer NJM, Arbous MS, Gommers DAMPJ, Kant KM, et al. Incidence of thrombotic complications in critically ill ICU patients with COVID-19. Thromb Res. 2020 Jul;191:145–147. doi: 10.1016/j.thromres.2020.04.013 32291094 PMC7146714

[9] Ventura-Díaz S, Quintana-Pérez JV, Gil-Boronat A, Herrero-Huertas M, Gorospe-Sarasúa L, Montilla J, et al. A higher D-dimer threshold for predicting pulmonary embolism in patients with COVID-19: a retrospective study. Emerg Radiol. 2020 Oct 6:1–11. doi: 10.1007/s10140-020-01859-1 Epub ahead of print. 33025219 PMC7538266

[10] Jin C, Tian C, Wang Y, Wu CC, Zhao H, Liang T, et al. A Pattern Categorization of CT Findings to Predict Outcome of COVID-19 Pneumonia. Front Public Health. 2020 Sep 18;8:567672. doi: 10.3389/fpubh.2020.567672 33072703 PMC7531052

[11] Myall KJ, Mukherjee B, Castanheira AM, Lam JL, Benedetti G, Mak SM, et al. Persistent Post-COVID-19 Inflammatory Interstitial Lung Disease: An Observational Study of Corticosteroid Treatment. Ann Am Thorac Soc. 2021 Jan 12. Epub ahead of print. doi: 10.1513/AnnalsATS.202008-1002OC 33433263 PMC8086530

[12] Townsend L, Fogarty H, Dyer A, Martin-Loeches I, Bannan C, Nadarajan P, et al. Prolonged elevation of D-dimer Levels in Convalescent COVID-19 patients is Independent of the Acute Phase Response. J Thromb Haemost. 2021 Feb 15. doi: 10.1111/jth.15267 Epub ahead of print. 33587810 PMC8013297

[13] Cosmi B, Legnani C, Cini M, Favaretto E, Palareti G. D-dimer and factor VIII are independent risk factors for recurrence after anticoagulation withdrawal for a first idiopathic deep vein thrombosis. Thromb Res. 2008;122(5):610–7. doi: 10.1016/j.thromres.2007.12.024 18304616

[14] Jara-Palomares L, Solier-Lopez A, Elias-Hernandez T, Asensio-Cruz MI, Blasco-Esquivias I, Sanchez-Lopez V, et al. D-dimer and high-sensitivity C-reactive protein levels to predict venous thromboembolism recurrence after discontinuation of anticoagulation for cancer-associated thrombosis. Br J Cancer. 2018 Oct;119(8):915–921. doi: 10.1038/s41416-018-0269-5 30318508 PMC6203717

[15] Dhont S, Derom E, Van Braeckel E, Depuydt P, Lambrecht BN. The pathophysiology of ´happy´hypoxemia in COVID-19. Respir Res 2020. 21(1): 198. doi: 10.1186/s12931-020-01462-5 32723327 PMC7385717

[16] Carlino MV, Valenti N, Cesaro F, Costanzo A, Cristiano G, Guarino M, et al. Predictors of Intensive Care Unit admission in patients with coronavirus disease 2019 (COVID-19). Monaldi Arch Chest Dis. 2020 Jul 15;90(3) 10.4081/monaldi.2020.1410 32672430

[17] Spyropoulos AC, Levy JH, Ageno W, Connors JM, Hunt BJ, Iba T, et al. Subcommittee on Perioperative, Critical Care Thrombosis, Haemostasis of the Scientific, Standardization Committee of the International Society on Thrombosis and Haemostasis. Scientific and Standardization Committee communication: Clinical guidance on the diagnosis, prevention, and treatment of venous thromboembolism in hospitalized patients with COVID-19. J Thromb Haemost. 2020 Aug;18(8):1859–1865. doi: 10.1111/jth.14929 32459046 PMC7283841

